# Differences in Fcgamma receptor IIa genotypes and IgG subclass pattern of anti-malarial antibodies between sympatric ethnic groups in Mali

**DOI:** 10.1186/1475-2875-7-175

**Published:** 2008-09-15

**Authors:** Elisabeth Israelsson, Manijeh Vafa, Bakary Maiga, Anna Lysén, Nnaemeka C Iriemenam, Amagana Dolo, Ogobara K Doumbo, Marita Troye-Blomberg, Klavs Berzins

**Affiliations:** 1 Department of Immunology, Wenner-Gren Institute, Stockholm University, Svante Arrheniusväg 16, SE-10691, Stockholm, Sweden; 2 Malaria Research and Training Center (MRTC), Faculty of Medicine and Pharmacy, University of Bamako, Bamako, Mali; 3 Department of Medical Microbiology & Parasitology, College of Medicine, University of Lagos, Nigeria

## Abstract

**Background:**

The Ig Fc receptor family is an important link between the humoral and cellular immune systems. The association of a dimorphism in amino acid 131 (R/H) of the FcγRIIa with malaria severity, the R-allele being associated with a milder disease outcome, led to the investigation of the possible impact of this polymorphism in the interethnic difference in malaria susceptibility seen between the Fulani and Dogon in Mali.

**Methods:**

Plasma from individuals from Mali (164 Fulani and 164 Dogon) were analysed for malaria-reactive and total IgG subclass antibodies using ELISA, and the same individuals were also genotyped for the FcγRIIa R131H polymorphism using RFLP-PCR. Statistical analyses of the IgG subclass levels were done by unpaired t-test and ANOVA, and genotype differences were tested by χ^2^-test.

**Results:**

While the two ethnic groups showed a similar frequency of the FcγRIIa 131 R/H heterozygote genotype, 131R/R dominated over the 131 H/H genotype in the Dogon whereas the Fulani presented a similar frequency of the two homozygote genotypes. The two alleles were evenly distributed in the Fulani, while the Dogon were clearly biased towards the R-allele. The Fulani showed higher levels of anti-malarial IgG1, -2 and -3 antibodies, with a higher proportion of IgG2, than the Dogon. In the Fulani, H-allele carriers had higher anti-malarial IgG2 levels than R/R homozygotes, while in the Dogon, the R-allele carriers showed the higher IgG2 levels. For anti-malarial IgG3, the R-allele carriers in the Fulani had higher levels than the H/H homozygotes.

**Conclusion:**

Taken together, the results showed marked interethnic differences in FcγRIIa R131H genotypes. Furthermore, the results indicate that the FcγRIIa R131H genotype may influence the IgG subclass responses related to protection against malaria, and that IgG2 may be of importance in this context.

## Background

Malaria remains one of the most prevalent infectious diseases in the world today, with high morbidity and mortality, and no effective control strategies. A rational vaccine design is limited by the lack of knowledge of how the immune system clears the infection. Several studies relate high titers of anti-malarial IgG to protection from severe malaria, and seroepidemiological studies from different endemic areas have demonstrated an association between IgG antibodies of the cytophilic subclasses IgG1 and IgG3 with protection against *Plasmodium falciparum *malaria [1,2]. In other study populations, IgG2 antibodies to certain *P. falciparum *antigens, have been shown to be related to protection [3,4], indicating that the role of the different IgG subclasses in malaria protection still needs to be clarified.

The Fcγ receptors on monocytes and other leukocytes are important structures in the immune defence against pathogens. The binding of antibodies to the Fc-receptors provokes important biological functions, e.g. antibody dependent cell-mediated cytotoxicity (ADCC) or inhibition (ADCI) and phagocytosis [5]. In humans, there are three families of Fc-receptors binding IgG, FcγRI (CD64), -RII (CD32) and -RIII (CD16). FcγRI is a high-affinity receptor that binds monomeric IgG, FcγRII and -RIII are low-affinity receptors, only binding complexed or aggregated IgG. Based on a single nucleotide polymorphism (SNP) in the FcγRIIa gene (G494A) (rs1801274), this receptor has two co-dominantly expressed allotypes, differing at amino acid position 131 (R/H). FcγRIIa-131H is the only human FcγR that binds IgG2 efficiently [6]. This polymorphism appears to affect the regulation of the IgG subclass production or turnover in humans [7], which could be a contributing factor to the inconclusive results seen in previous studies on the FcγRIIa-R131H polymorphism in relation to malaria. Some of the studies are associating the FcγRIIa-131R/R genotype with protection against malaria, and the FcγRIIa-131H/H genotype with susceptibility to the disease (reviewed by Braga [8]). However, recent publications suggest the FcγRIIa-131H allele to be associated to protection against severe malaria [9,10]. This discrepancy is still not understood; it may be caused by different genetic backgrounds or by differences in the pathogen pressure.

Several studies have demonstrated differences in susceptibility to malaria between different ethnic groups. In West Africa, the Fulani were shown to have a lower incidence of malaria than other sympatric groups, despite the same exposure and no differences in socio-cultural circumstances [11]. Studies in Burkina Faso extended those results, demonstrating that the Fulani are less parasitized, less affected by malaria and have higher anti-malarial immune responses than the sympatric Mossi and Rimaibé groups [12]. This is also true for the Malian Fulani and their sympatric neighbours, the Dogon [13]. Previous results have shown that this relative resistance to malaria in the Fulani, as compared to other sympatric tribes, appears to be pathogen related and not due to a general hyper-reactive immune system [14]. Various markers confirm that the Fulani are genetically distinct from other African tribes, and it was thus proposed that established malaria resistance factors would be at a higher frequency in the Fulani, but the reverse was found [13,15].

In an attempt to further define potential genetical and immunological factors contributing to these ethnic differences in malaria susceptibility, the FcγRIIa genotypes in sympatric individuals from Mali were analysed and related to both the total and the malaria reactive IgG subclass profiles.

## Materials and methods

### Study area

The study area is located in the Mopti area about 850 km Northeast of Bamako, the capital of Mali. Four villages (Mantéourou, Naye, Binédama, and Anakédié) were identified for the study. The Mantéourou and Naye are divided into two subdivisions, Mantéourou Peulh/Mantéourou Dogon and Naye Peulh/Naye Dogon-Dinsogou. The two subdivisions are separated by 300–500 meters and inhabited either by Fulani or Dogon. The other two villages, Binédama and Anakédié, are exclusively populated by either Fulani or Dogon, respectively, and are located approximately 1,000 meter apart. Malaria transmission is mesoendemic in the area, with *P. falciparum *as the main parasite species. The entomological inoculation rate (EIR) is similar in both ethnic groups [13]. In this area the dry season extends from October to May and the rainy season from July to October.

### Human samples

Blood was collected during the rainy season in September 2005 from 328 (164 Fulani and 164 Dogon) asymptomatic, randomly selected donors, aged 1–60 years. For PCR analyses, finger prick blood was collected on filter papers. For plasma samples, either finger prick blood was collected in capillaries or venous blood in vacutainer tubes, both with heparin as anti-coagulant. Spleen enlargement was established based on palpation and graded on the Hackett score (0–5). The proportion of individuals that had enlarged spleens was denoted spleen rate. Informed consent was obtained from all participants or their guardians and it was performed in two steps; first an orally community consent is obtained prior to the study, where the whole community is informed of the aim of the study. Second an individual consent is obtained before blood collection; an alphabetized volunteer sign the form and a non-alphabetized volunteer puts his fingerprint. The Ethical committee of the Faculty of Medicine and Pharmacy of Mali and the National Ethics Committee in Sweden approved the study.

### Plasma preparation

The capillaries were cut, and blood cells and plasma were separated by centrifugation. The plasmas were stored in -20°C.

### DNA preparation

DNA was extracted from filter papers using Chelex-100, and then stored at -20°C. In brief, discs of the same size were cut out and incubated overnight in 1 ml of 0.5% saponin in phosphate-buffered saline (PBS) at 4°C, and were then washed 15–30 minutes in 1 ml PBS at 4°C. The discs were then boiled in 200 μl of 5% Chelex-100 in water for 15 minutes, and the DNA was collected in supernatants after centrifugation at 8,000 × g for 3 minutes.

### Crude malaria antigen

*Plasmodium falciparum *parasites (strain F32) were maintained in continuous cultures as described earlier [16] and kept synchronized by repeated treatment with sorbitol [17]. When the parasitaemia in the cultures was 10% or more, late stage parasites were isolated on 60% Percoll, sonicated and used as antigen in the ELISA [18].

### Enzyme-linked immunosorbent assay (ELISA)

EIA/RIA plates (Costar, Corning, NY) were coated overnight at 4°C with crude *P. falciparum *antigen in sodium carbonate buffer (pH 9.6) at the concentration 10 μg/ml. After blocking at 37°C with carbonate buffer containing 1% BSA (w/v) for 2 hours, the plates were washed with saline containing 0.05% Tween 20. Serum dilutions (50 μl, 1:500 for IgG1 and IgG3 and 1:50 for IgG2 and IgG4) were added to the plates and incubated 1 h at 37°C. To detect IgG2, IgG3 and IgG4, 50 μl biotin-conjugated mouse anti-human monoclonal antibodies (IgG2: clone G18-21, 1:3000 dilution, PharMingen, San Diego, CA. IgG3: clone HP6047, 1:1000 dilution, Caltag Laboratories, Burlingame, CA. IgG4: clone HP6025, 1:2000 dilution, Sigma, St. Louis, MO) and alkaline phosphatase conjugated streptavidine (Mabtech AB, Sweden) were used. Detection of IgG1 was done with a monoclonal mouse anti-human IgG1 antibody (50 μl of 1:1000 dilution, clone NL16-purified, SkyBio Limited, Bedfordshire, U.K) and alkaline phosphatase conjugated goat anti-mouse Ig (Dako Denmark A/S, Glostrup, Denmark). The assay was developed with p-nitrophenyl phosphate disodium salt (Sigma, St. Louis, MO) as substrate and the optical densities were read at 405 nm in ELISA plate reader (VmaxTM Kinetic Microplate Reader, Menlo Park, CA). The concentrations of anti-malarial antibodies, expressed as ELISA-units (a μg/ml equivalent), were calculated from standard curves obtained in a sandwich ELISA with six dilutions of myeloma proteins of the IgG1-4 isotypes (Serotec, Oxford, UK). No cross reactivity between the different subclass reagents were detected in this system.

The total concentrations of IgG subclass 1–4 were determined with PeliClass human IgG subclass kit (Sanquin Reagents, Amsterdam, The Netherlands). In short, precoated micro titre plates where incubated with diluted sera (1:30 000 for IgG2, IgG3 and IgG4 and 1:240 000 for IgG1) at 37°C for 1 h. For detection, an HRP-conjugated anti-human IgG antibody was used and the assay was developed with ABTS-substrate and read at 405 nm in ELISA plate reader (VmaxTM Kinetic Microplate Reader, Menlo Park, CA).

### RFLP analysis of the FcγRIIa polymorphism

The *R131H *FcγRIIa polymorphism (rs1801274) was investigated using restriction fragment length polymorphism (RFLP) analysis as described by Jiang *et al *[19]. The PCR amplification was performed in 10 μl reactions using 2.5 μl of genomic DNA, 5 μl of 2× ready-to-use PCR Master Mix (ABgene, Surrey, UK.) and 0.25 μl of each primer (Forward: 5'-GGAAAATCCCAGAAATTCTCGC-3' and Reverse: 5'-CAACAGCCTGACTACCTATTACGCGGG-3', (MWG-Biotech AG, Ebersberg, Germany)). The PCR was carried out in an Eppendorf Mastercycler^® ^(Eppendorf AG, Hamburg, Germany) using a 10 min denaturation at 94°C followed by 35 cycles with 94°C for 30 sec, 56°C for 30 sec and 72°C for 45 sec. The final extension was at 72°C for 5 min. Five μl of the PCR product were used for the enzymatic digestion with 2.5 U Bsh12361 (Fermentas GMBH, St Leon Rt, Germany) at 37°C over night. The resulting fragments (H/H: 343 bp, 23 bp, R/R: 322 bp, 44 bp, H/R: 343 bp, 322 bp, 44 bp, 23 bp) were analyzed on ethidium bromide stained 2.5% agarose gels.

### Parasite detection by PCR

In order to detect an ongoing *P. falciparum *infection and to determine the number of merozoite surface protein (*msp) 2 *(FC27 and 3D7) clones per infection, a nested PCR was performed. Amplifications were done in a 10 μl reaction using 2.5 μl of DNA, iProof™ High-Fidelity Master Mix (BIO-RAD Laboratories, Hercules, CA) and 500 nM of primer pairs corresponding to the outer conserved region of the polymorphic repetitive block 3 of *msp2*. DNA was denatured at 98°C for 1 min; then PCR was performed for 30 cycles of 98°C for 10 sec, 61°C for 20 sec and 72°C for 30 sec, with a final 5 min extension. One μl of the PCR product was re-amplified with primers specific for FC27 and IC/3D7 allelic types of *msp2*, using the following program: 30 sec at 98°C; 25 cycles of 98°C for 10 sec, 61°C for 20 sec and 72°C for 10 sec and a final extension for 5 min. The primer sequences were presented elsewhere [20]. The number of products, corresponding to the number of infecting FC27 and IC/3D7 clones, was counted after visualization on ethidium bromide (EtBr) stained 2.5 and 1.5% agarose gel, respectively.

### Hb AS genotyping

To detect HbAS carriers a PCR procedure was used. In short, the PCR amplification was performed in 10 μl reactions using 2 μl of genomic DNA, 5 μl of 2× ready-to-use PCR Master Mix (ABgene, Surrey, UK.) and 0.25 μl of each primer (Forward: 5'-ACACAACTGTGTTCACTAGC-3' and Reverse: 5'-CAACTTCATCCACGTTCACC-3', (MWG-Biotech AG, Ebersberg, Germany)). The PCR was carried out in an Eppendorf Mastercycler^® ^(Eppendorf AG, Hamburg, Germany) using a 5 min denaturation at 95°C followed by 35 cycles with 95°C for 30 sec, 56°C for 30 sec and 72°C for 30 sec. The final extension was at 72°C for 5 min. The entire PCR product were used for the enzymatic digestion with 4 U *Dde*I (Invitrogen) at 37°C for 2 h. The resulting fragments were analyzed on ethidium bromide stained 2.5% agarose gels.

### Statistical analysis

All the statistical analyses were performed in StatView version 5.0.1 if not stated otherwise. Differences in malariometric indexes between the two ethnic groups were tested with Mann-Whitney U-test. The observed genotype frequencies of the FcγRIIa R131H polymorphism in the two ethnic groups were tested for differences with χ^2^-test. Multiple regression analyses, with IgG subclasses as dependent variables and age, ethnicity, gender, parasite density and FcγRIIa R131H genotypes as independent variables, were performed to assess the influence on these variables on IgG subclass levels. Association analyses were performed in Unphased version 3.0.7.

As the antibody data did not show normal distribution, they were log-transformed, after which they all were normally distributed. Comparisons between the two ethnic groups were done using unpaired t-test. For analyses of genotype and IgG subclass levels, ANOVA was used. To determine differences between the populations, the Fisher's PLSD was used.

To test for potential ethnic differences, as well as for genotype-based differences in spleen rate, χ^2^-test was used. Ethnic and genotype-based differences in parasite density, numbers of parasite clones and haemoglobin levels were analysed by Mann-Whitney U-test or Kruskal-Wallis test.

A p-value of 0.05 or less was considered to be significant, and p-values between 0.05 – 0.1 were considered to be marginally significant. No correction for the number of tests was done, so there is a risk of presenting some overestimated significances.

## Results

### Study population

There were no differences in age and gender between the two study populations (Table 1). The parasite prevalence differed between the two ethnic groups, 58% being infected among the Dogon, compared to 43% among the Fulani (p = 0.008). Also, the number of circulating parasite clones differed between the groups, the Dogon having more parasite clones than the Fulani (5.6 ± 2.9 and 4.5 ± 2.7, respectively, p = 0.02) (Table 1). With regard to spleen rate, the Fulani had an enlarged spleen more frequently as compared to the Dogon (37% and 11%, respectively; p < 0.0001) (Table 1). The haemoglobin levels were found to be significantly higher in the Dogon group than in the Fulani (13.1 ± 2.4 and 10.6 ± 2.5, respectively, p < 0.0001) (Table 1). The frequencies of Hb AS were found to be similar in the two ethnic groups (7.2% in Dogon and 7.9% in Fulani) (Table 1).

**Table 1:** Malariometric indexes in the participating Fulani and Dogon communities in Mali

	Dogon	Fulani	P value^a^
	
Age-range (years)	1–58	1–60	
Age-median (years)	16	14.5	
Gender distribution (% women)	48	49	
Spleen rate^b ^(%)	11	37	**< 0.0001**
Parasite prevalence^c ^(%)	58	43	**0.008**
Number of clones^d ^± SD	5.63 ± 2.85	4.52 ± 2.7	**0.02**
Parasite density^e ^± SD	662 ± 3206	716 ± 2844	**ns**
Haemoglobin levels (mean ± SD)	13.1 ± 2.4	10.6 ± 2.5	**< 0.0001**
HbAS prevalence (%)	7.2	7.9	**ns**
	
Number of subjects	164	164	

^a ^Differences between the two ethnic groups tested by Mann-Whitney U test

^b ^Percentage of individuals with enlarged spleen

^c ^Proportion of infected individuals, measured by PCR

^d ^Mean number of clones identified as *msp2 *alleles, excluding PCR negatives

^e ^*P. falciparum *density (parasites/μl)-mean.

### FcγRIIa R131H genotyping

All individuals of the two study groups were genotyped for the G494A single nucleotide polymorphism (R131H) in the FcγRIIa gene (rs1801274). Both allele and genotype frequencies were found to be in Hardy-Weinberg equilibrium. While both ethnic groups showed a similar frequency of heterozygotes (131H/R), there was a statistically significant difference with regard to the frequency of homozygotes (Table 2). The 131R/R genotype was dominating among the Dogon, while the homozygote genotypes were evenly distributed in the Fulani (p = 0.0008). This genetic difference between the two ethnic groups was even more pronounced when comparing the allele frequencies, the 131H and 131R alleles being presented in equal frequencies in the Fulani and the 131R allele dominating in the Dogon (p < 0.0001) (Table 2). Moreover, weak associations were found between the H-allele and the Fulani ethnic group (OR = 0.73, p = 0.05) and the R-allele and the Dogon ethnic group (OR = 1.37, p = 0.05).

**Table 2:** Genotype and allele frequencies of FcγRIIa R131H polymorphism

Tribe	R/R	H/R	H/H	**P-value^a^**	R-allele	H-allele	**P-value^a^**
Dogon	65 (41%)	71 (44%)	24 (15%)	**0.0008**	201 (63%)	119 (37%)	**< 0.0001**
Fulani	39 (24%)	78 (48%)	47 (29%)		156 (48%)	172 (52%)	

^a ^Statistical analysis of differences in frequencies between the two ethnic groups tested with χ^2^-test.

### IgG subclass distribution

**Figure 1:**
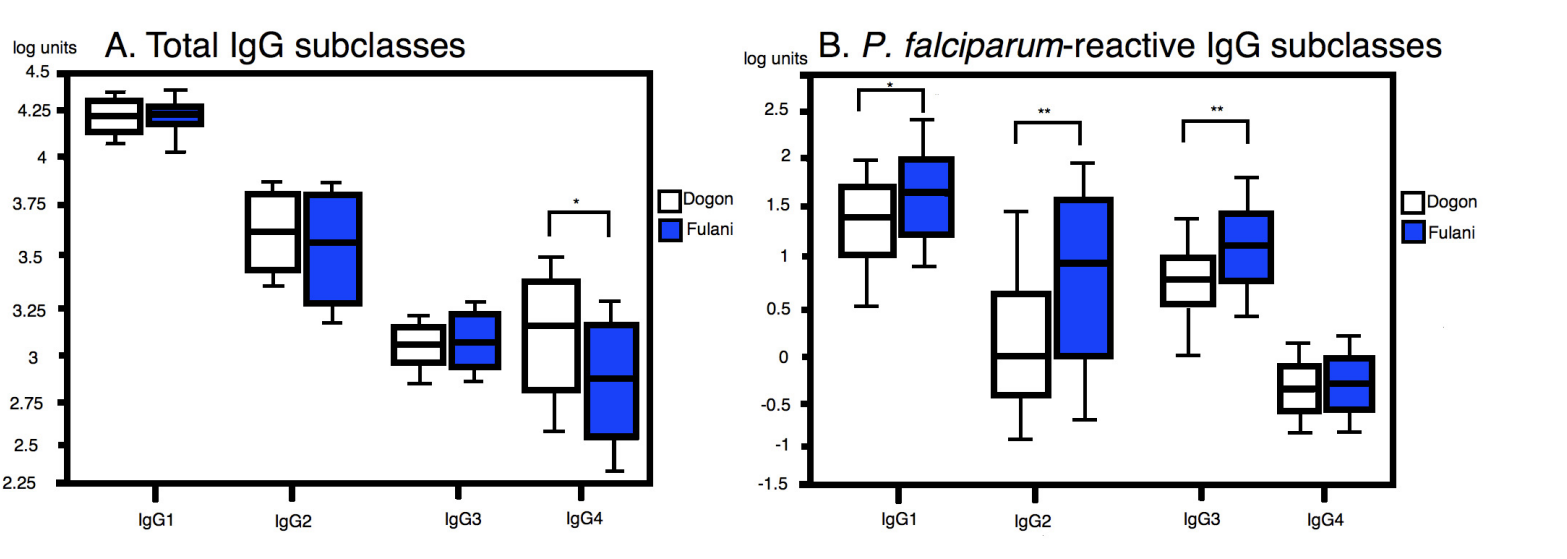
**The distribution of total IgG subclasses (A), and *P. falciparum*-reactive IgG subclasses (B), as assessed by ELISA.** The results are expressed in ELISA units and the values are represented as the log 10 values of the concentration of each IgG subclass. The boxes represent the values between the 25% to the 75% quartile and the line indicates the median. The whiskers indicate the 10% and the 90% quartile. Statistical analysis done by unpaired t-test. * P < 0.05, ** P < 0.0001.

Possible differences in relation to ethnicity or FcγRIIa genotypes and patterns of total- or anti-malarial IgG subclasses were analysed. All IgG subclasses, except for IgG4, increased in concentration with age for both ethnic groups. For total IgG subclass levels, the Dogon showed slightly higher IgG4 concentrations than the Fulani (p = 0.03), while none of the other subclasses showed any difference between the two ethnic groups (Figure 1A).

For the anti-malarial IgG antibody subclasses, the Fulani showed significantly higher levels of IgG1 (p = 0.0003), IgG2 and IgG3 (p < 0.0001, respectively). For anti-malarial IgG4, both ethnic groups showed very low levels and no difference between the ethnic groups was seen (p = 0.2). (Figure 1B). The most dominant subclass among the anti-malarial antibodies was IgG1, followed by IgG3, IgG2 and IgG4 in both ethnic groups. However, some Fulani individuals showed a stronger IgG2 than IgG3 response, hence, for those individuals the pattern was IgG1 followed by IgG2, IgG3 and IgG4 (Figure 1B.). Comparison between the two ethnic groups, of what proportion of the total IgG subclasses were represented by anti-malarial antibodies, showed that the Fulani group had a significantly higher percentage of anti-malarial antibodies than the Dogon for all IgG subclasses, except for IgG4 (Table 3).

**Table 3:** Geometric mean percentage of *P. falciparum *reactive IgG subclasses among the total IgG subclass concentration.

	Specific IgG subclasses/total IgG subclasses (%)			
	
	IgG1	IgG2	IgG3	IgG4
	
Dogon	2.4 ± 1.2	1.8 ± 1.5	1.8 ± 1.3	0.7 ± 1.5
Fulani	3.2 ± 1.2	2.6 ± 1.4	2.4 ± 1.7	0.8 ± 1.4
**p-value^a^**	**< 0.0001**	**0.0005**	**< 0.0001**	0.11

Data are presented as geometric mean percentages (the mean percentages of the logged values) ± SD.

^a ^Statistical analysis of differences between the two ethnic groups for each IgG subclass tested with t-test.

The patterns of the different IgG subclasses among the anti-malarial antibodies and total IgGs were compared between the two ethnic groups, analysing the ratios of IgG1/IgG2, IgG1/IgG3 and IgG2/IgG3 (Table 4). IgG4 was excluded from this analysis, since the levels of anti-malarial IgG4 were consistently low in both groups. While the ratios for anti-malarial IgG1/IgG3 did not differ between the two groups (p = 0.3), the ratios including IgG2 indicated that the Fulani have a more IgG2 directed response than Dogon (IgG1:IgG2 and IgG2:IgG3, p < 0.0001, respectively). This was not reflected in the ratios regarding total IgG subclass levels, where no differences between the two ethnic groups were seen.

**Table 4:** Ratio between IgG subclasses, both *P. falciparum *reactive and total.

	*P. falciparum *specific IgG subclasses			Total IgG subclasses		
	IgG1:IgG2	IgG1:IgG3	IgG2:IgG3	IgG1:IgG2	IgG1:IgG3	IgG2:IgG3
	
Dogon	4.3 : 1	4.9 : 1	1.1 : 1	4.3 : 1	13.6 : 1	3.1 : 1
Fulani	2.5 : 1	4.6 : 1	1.8 : 1	3.6 : 1	13 : 1	3.6 : 1

**p-value^a^**	**< 0.0001**	0.28	**< 0.0001**	0.2	0.26	0.48

Data are presented as the mean ratio.

^a ^Statistical analysis of differences in ratios between the two ethnic groups tested with t-test.

### FcγRIIa R131H and distribution of *P. falciparum*-reactive IgG subclasses

In order to see if the FcγRIIa R131H genotype could have an effect on the *P. falciparum*-reactive IgG subclass levels, multiple regression analyses with the different IgG subclass antibody levels as dependent variables were performed. When adjusted for age, gender, ethnicity and parasite density, the FcγRIIa R131H genotype showed a weak but significant predictive effect on *P. falciparum*-reactive IgG3 levels (adjusted R^2 ^= 0.324 and p = 0.027) with higher levels in individuals homozygous for the R-allele, and a non-significant effect on total IgG1 levels (adjusted R^2 ^= 0.117 and p = 0.09), with higher IgG1 levels in HH carriers. When separating the two ethnic groups in the analyses, the FcγRIIa R131H genotype showed a tendency for a predictive effect on *P. falciparum*-reactive IgG2 levels in both Fulani and Dogon (adjusted R^2 ^= 0.266 and p = 0.08 and adjusted R^2 ^= 0.350 and p = 0.06, respectively). Higher antibody levels where found in HH carriers in the Fulani, while RR carriers had higher levels in the Dogon,

The total IgG1 levels in the Fulani were significantly but still weakly predicted by the FcγRIIa R131H genotype (p = 0.04).

In order to further decipher the possible influence of the FcγRIIa R131H genotypes on IgG subclass levels, comparisons of the levels of the different IgG subclasses among the *P. falciparum*-reactive antibodies between the different FcγRIIa R131H genotypes were performed. Overall genotype differences regarding both IgG2 and IgG3 levels were seen for both ethnic groups, as well as a difference in IgG4 levels in the Fulani ethnic group (Table 5). For the Dogon, the H/H individuals showed a lower anti-malarial IgG2 level than both H/R and R/R carriers (p = 0.02), while the H/R individuals had lower levels of *P. falciparum*-reactive IgG3, than the R/R individuals (p = 0.03). For the Fulani, the R/R individuals showed lower IgG2 concentrations than the H/R subjects (p = 0.05), but there was also a tendency for H/H individuals to present higher IgG2 levels than R/R genotype carriers. The concentrations of IgG3 were lower in the H/H individuals compared to the H/R heterozygotes (p = 0.01), but there was also a tendency for lower levels as compared to R/R subjects. For anti-malarial IgG4 concentrations, the H/H homozygotes had lower levels than H/R individuals (p = 0.04).

**Table 5:** IgG subclass levels separated by FcγRIIa R131H genotypes and alleles in the Fulani and Dogon ethnic groups

	Dogon						Fulani					
	
	no.	age	IgG1	IgG2	IgG3	IgG4	no.	age	IgG1	IgG2	IgG3	IgG4
	
H/H	24	15	15 ± 2.9	0.4 ± 5.6	4.5 ± 3.5	0.54 ± 2.0	39	15	34 ± 4.4	5.9 ± 11	6.3 ± 2.5	0.29 ± 7.8
H/R	69	16	17 ± 3.9	1.2 ± 6.7	3.8 ± 2.6	2.3 ± 2.3	68	23	38 ± 3.7	7.5 ± 9.1	12 ± 2.8	1.8 ± 2.6
R/R	64	20	24 ± 3.5	1.5 ± 8.1	6.2 ± 2.9	0.41 ± 2.9	36	21	24 ± 3.4	2.4 ± 9.8	9.5 ± 2.9	0.42 ± 2.3
**p-value^a^**		0.07	0.2	**0.02**	**0.03**	0.5		0.07	0.2	**0.05**	**0.01**	**0.04**

R-allele carriers	133	18	20 ± 3.7	1.3 ± 7.3	4.8 ± 2.8	0.43 ± 2.5	93	22	33 ± 3.6	5.0 ± 10	11 ± 2.8	0.5 ± 2.5

**p-value^b^**		0.3	0.4	**0.007**	0.8	0.2		0.03	0.08	0.1	**0.009**	0.4

H-allele carriers	104	22	17 ± 3.6	0.89 ± 6.7	3.9 ± 2.8	0.46 ± 2.3	107	20	37 ± 3.9	6.8 ± 9.5	9.4 ± 2.8	0.44 ± 4.4

**p-value^c^**		0.02	0.9	0.7	**0.004**	**0.02**		0.9	0.09	**0.02**	0.9	0.8

The results are expressed in ELISA units and presented as geometric means ± SD.

^a ^Statistical analysis of differences in IgG subclass levels between the different genotypes tested with ANOVA

^b ^Statistical analysis of differences in IgG subclass levels between the R-allele carriers and HH homozygotes Mann-Whitney.

^c ^Statistical analysis of differences in IgG subclass levels between the H-allele carriers and RR homozygotes by Mann-Whitney.

Analysis of the R-allele carriers in comparison with the H/H homozygotes revealed that there was only a difference regarding IgG3 in the Fulani (Table 6), with R-allele carriers having higher mean levels of anti-malarial IgG3 than H/H homozygotes (p = 0.004). The Dogon, on the other hand, only showed a statistically significant difference regarding IgG2, with R-allele carriers having higher mean levels of anti-malarial IgG2 than H/H homozygotes (p = 0.007). A comparison between H-allele carriers and R/R homozygotes (Table 6) showed a significant difference in malaria specific IgG3 levels for Dogon (p = 0.009), with H-allele carriers having lower levels than R/R homozygotes. The Fulani showed a significant difference regarding IgG2, where the H-allele carriers had higher levels than R/R homozygotes (p = 0.02).

**Table 6:** Malariometric indexes separated by FcγRIIa R131H genotypes in the Fulani and Dogon ethnic groups.

	Dogon				Fulani			
	
	HH	HR	RR	**p-value^a^**	HH	HR	RR	**p-value ^a^**
	
Age (mean ± sd)	15 ± 12	16 ± 13	20 ± 13		15 ± 13	23 ± 18	20 ± 17	
Spleen rate ^b ^(%)	8	13	11	0.8	38	36	36	0.9
Hb (mean ± sd)	13 ± 2	12.6 ± 2.4	13.3 ± 2.6	0.4	11 ± 2	10.6 ± 2.7	10.8 ± 2.3	0.9
Parasite prevalence ^c ^(%)	67	58	60	0.7	49	33	59	**0.02**
Parasite density ^d ^(mean ± sd)	907 ± 4138	546 ± 1803	243 ± 843	0.5	330 ± 1138	530 ± 2155	1533 ± 4734	0.2
Clones ^e ^(mean ± sd)	4.0 ± 3.7	2.9 ± 3.3	3.6 ± 3.7	0.4	1.9 ± 2.6	1.3 ± 2.5	3.3 ± 3.5	**0.006**
HbAS ^f ^(%)	0	10	6	0.2	6	3	18	**0.01**

^a ^Differences between the three different genotypes.

^b ^Percentage of individuals with enlarged spleen

^c ^Proportion of infected individuals, measured by PCR

^d ^*P. falciparum *density (parasites/μl)-mean

^e ^Mean number of clones identified as *msp2 *alleles, excluding PCR negatives

^f ^Frequency of HbAS carriers detected by PCR.

### FcγRIIa R131H and malariometric data

In order to see if the FcγRIIa R131H genotypes affected haemoglobin levels, parasite density, number of circulating parasite clones, frequency of Hb AS and spleen rate, analyses of these parameters within each genotype were performed. No differences were found for parasite density, haemoglobin levels or spleen rate. The number of circulating parasite clones differed in the Fulani group, where individuals with the RR genotype had higher numbers of clones than HR and HH genotype individuals (mean ± SD: 3.3 ± 3.5, 1.3 ± 2.5 and 1.9 ± 2.6, respectively, p = 0.006) (Table 6). Also, the frequency of Hb AS carriers differed between the genotypes in the Fulani group, again RR individuals having a higher Hb AS frequency than HR and HH individuals (p = 0.01, Table 6). When comparing the parasite prevalence, it was found that the Fulani HR carriers had a higher frequency of parasite positivity than the other genotypes (Table 6). Furthermore, when comparing only the parasite positive, a difference in distribution pattern was found between the ethnic groups. While the Fulani showed an even distribution of parasite positive individuals between the genotypes, the Dogon had a lower frequency of parasite positive individuals with the HH genotype than in HR and RR (Fulani: HH: 32%, HR: 36%, RR: 32%, Dogon: HH: 17%, HR: 43%, RR: 40%. p = 0.066), but this difference in patterns did not reach statistical significance.

## Discussion

This study shows that two sympatric ethnic groups in Mali, the Fulani and the Dogon, exhibit differential frequencies in the expression of FcγRIIa R131H genotypes and allotypes. It furthermore, confirms and extends the previous findings regarding differences in anti-malarial responses between sympatric ethnic groups living in West Africa [12,13]. The Fulani were less parasitized, had fewer parasite clones and had higher anti-malarial IgG subclass levels than the sympatric ethnic group, the Dogon. Fulani also showed a higher spleen rate as compared to Dogon. Interestingly, the Fulani showed significantly lower haemoglobin levels as compared to the Dogon, which was recently confirmed in both a longitudinal study and two cross sectional studies in the same study area (Dolo *et al*, personal communication).

The FcγRIIa R131H genotype results of this study contradict those from previous reports, where the R/R genotype has been associated with protection (reviewed by Braga [8]). Here it was revealed that the H/H genotype is more common in the Fulani, who are considered to be less susceptible to malaria, and this was recently confirmed by a study in Sudan [21], where the H allele was more common in the Fulani group than in the sympatric Masaleit group. In line with these findings, others have recently reported that the H/H genotype is related to protection [9,10,22], suggesting that the H/H genotype might contribute to the relative protection from malaria in the Fulani. The influence of the FcγRIIa R131H polymorphism on the relative susceptibility to malaria, could in part be due to the association of this polymorphism with a differential IgG subclass production or turnover [7]. In line with this, the present study shows an association between differential levels of IgG1 and IgG3 antibodies and FcγRIIa R131H genotypes. The previous suggestion, that the presence of the R-allele is an important factor in protection from severe disease [23] is compatible with the finding in this study that the Fulani had higher levels of anti-malarial IgG3 among the R-allele carriers than among the H/H individuals. Also the Dogon group showed higher IgG3 antibody levels among R/R homozygotes than the H-allele carriers. In individuals from the Dogon ethnic group, the R-allele also seems to be related to higher IgG2 levels, while in Fulani, the H-allele carriers have higher IgG2 levels. Why the two ethnic groups have different distribution of the IgG2 subclass is not known. However, it may be speculated that since the FcγRIIa 131R receptor is being less effective in binding IgG2, more IgG2 will be present free in the circulation of individuals with the R-allele than those with the H-allele. This could explain the higher IgG2 levels found in Dogon individuals carrying the R-allele, but not the higher IgG2 levels in the Fulani individuals carrying the H-allele. Interestingly, the Fulani show a FcγRIIa genotype frequency similar to what has previously been reported for Caucasians [24,25], while the Dogon have the same H/H frequency as African-Americans [25], but differs regarding R/R and R/H genotype frequency. Since the Fulani have been shown to be closer to Caucasians than Africans for some genetic markers [26], the difference seen between the two ethnic groups regarding FcγRIIa appears therefore not to have been driven by malaria pressure, but rather be a reflection of their different genetic background.

The impact of the FcγRIIa R131H polymorphism on the clinical outcome of malaria has been shown in several studies [3,4,9,10,22,23,27-29]. In this study, however, the analyses of a possible effect of the polymorphism on some malariometric factors (age, spleen rate, haemoglobin levels, parasite density, number of clones and carriage of Hb AS) showed that only the frequency of Hb AS and the number of circulating clones differed, with H-allele carriers having a lower Hb AS frequency and fewer clones than R/R homozygotes, but only in the Fulani group (Table 6). It has been indicated that the number of clones is related to protection against severe malaria [30], and together with the present results, it appears that the H-allele might be a prognostic factor in this context. It is important to notice that this parameter fluctuates considerably in individuals [31] and that it is difficult to conclude anything based on only one measurement. The unequal balance in the distribution of parasite positive individuals between the genotypes found in the Dogon, where the HH homozygotes had fewer positive individuals than the HR and RR genotype groups, may suggest that the HH genotype could be more protective due to the pattern of fewer parasite positive individuals in this group.

No differences between the two ethnic groups were shown when comparing total IgG subclasses, except for IgG4, were the Dogon showed slightly higher concentrations than the Fulani. This confirms the previous suggestion, that the relative resistance seen in the Fulani as compared to other sympatric ethnic groups appears to be pathogen related, and not a result of a generally more activated immune system in the Fulani [14].

Regarding anti-malarial IgG subclasses, the Fulani had higher levels of all subclasses, except for IgG4, which was very low in both ethnic groups. When calculating the percentage of anti-malarial IgG subclass antibodies within the total IgG concentrations, the Fulani showed a higher percentage of all IgG subclasses of anti-malarial antibodies, except for IgG4, where there were no differences between the two ethnic groups. For the ratios between IgG1:IgG2, IgG1:IgG3 and IgG2:IgG3, the Fulani had higher IgG1, IgG2 and IgG3 levels, while the Dogon had predominant IgG1 and IgG3 responses. Previous studies suggest that IgG1 and IgG3 are the most important IgG subclasses in the protection against malaria [1]. IgG1 and IgG3 are known to opsonate infected red blood cells, while IgG2 and IgG4 have been shown to inhibit this opsonization. [32]. However, other reports suggest a protective role of anti-malarial IgG2 [32], and high IgG2 and low IgG4 levels of anti-malarial antibodies have been associated with resistance to *P. falciparum *malaria [3]. The data reported here showed that the Fulani have higher anti-malarial IgG2 levels compared to the Dogon, while IgG4 were similarly low in the two ethnic groups. This suggests that the association of high IgG2 and low IgG4 levels reported by Aucan *et al *[3] could be a contributing factor for the relative resistance to malaria seen in the Fulani. However, even though the results presented here do not contradict this suggestion, the IgG4 concentrations were overall very low in this study and were therefore excluded from the analyses. Thus, the role of IgG4 in the ethnic differences in malaria susceptibility seen between the Fulani and the Dogon of Mali remains to be investigated.

Carriage of haemoglobin AS has been reported to influence the anti-malarial IgG2 concentrations in Gabonese children, which was independent of the FcγRIIa R131H genotype [4]. Preliminary observations did not detect any influence of carriage of haemoglobin AS on anti-malarial IgG2 levels in this study. No differences in frequency of AS carriers between the ethnic groups were seen (7.2% in Dogon and 7.9% in Fulani), and there was no difference in levels of anti-malarial IgG2 between the AS and AA genotype carriers.

## Conclusion

In conclusion, the relative resistance to malaria seen in the Fulani as compared to the Dogon appears not to be a generally more reactive immune system, but rather a pathogen related phenomenon. Furthermore, the results indicate that the FcγRIIa 131H allele may be associated with a lower susceptibility to malaria in the Fulani group as compared to other sympatric ethnic groups, and also that the FcγRIIa R131H polymorphism may have an influence on the IgG subclass responses and thereby affecting the malaria susceptibility.

## Competing interests

The authors declare that they have no competing interests.

## Authors' contributions

EI contributed to the design of the study, performed the FcγRIIa genotyping and the ELISA, carried out the statistical analyses and drafted the manuscript. MV carried out the parasite genotyping. BM examined all study subjects in Mali and collected all clinical data. AL contributed to the FcγRIIa genotyping and the ELISA. NI performed the sickle cell analyses. AD and OKD were responsible for clinical data in Mali. KB designed the study. MTB and KB revised the manuscript and financed and supervised the research. All authors read and approved the final manuscript.
